# Essential roles of zebrafish *rtn4*/Nogo paralogues in embryonic development

**DOI:** 10.1186/1749-8104-9-8

**Published:** 2014-04-23

**Authors:** Alejandro Pinzón-Olejua, Cornelia Welte, Houari Abdesselem, Edward Málaga-Trillo, Claudia AO Stuermer

**Affiliations:** 1 Department of Biology, University of Konstanz, Universitätsstrasse 10, 78476 Konstanz, Germany; 2 Department of Immunology and Microbiology, Weill Cornell Medical College in Qatar, Education City, Qatar Foundation, Huwar Street, 24144 Doha, Qatar; 3 Universidad Peruana Cayetano Heredia, Facultad de Ciencias y Filosofía, Laboratorios de Investigación y Desarrollo, Av. Honorio Delgado 430, Lima 31, Perú

**Keywords:** Brain and spinal cord development, Larval motility, Morpholino knockdown, Nogo, Reticulon, *rtn4*, Zebrafish

## Abstract

**Background:**

As a consequence of gene/genome duplication, the *RTN4*/Nogo gene has two counterparts in zebrafish: *rtn4a* and *rtn4b*. The shared presence of four specific amino acid motifs—M1 to M4—in the N-terminal region of mammalian RTN4, and zebrafish Rtn4b suggests that Rtn4b is the closest homologue of mammalian Nogo-A.

**Results:**

To explore their combined roles in zebrafish development, we characterized the expression patterns of *rtn4a* and *rtn4b* in a comparative manner and performed morpholino-mediated knockdowns. Although both genes were coexpressed in the neural tube and developing brain at early stages, they progressively acquired distinct expression domains such as the spinal cord (*rtn4b*) and somites (*rtn4a*). Downregulation of *rtn4a* and *rtn4b* caused severe brain abnormalities, with *rtn4b* knockdown severely affecting the spinal cord and leading to immobility. In addition, the retinotectal projection was severely affected in both morphants, as the retina and optic tectum appeared smaller and only few retinal axons reached the abnormally reduced tectal neuropil. The neuronal defects were more persistent in *rtn4b* morphants. Moreover, the latter often lacked pectoral fins and lower jaws and had malformed branchial arches. Notably, these defects led to larval death in *rtn4b*, but not in *rtn4a* morphants.

**Conclusions:**

In contrast to mammalian Nogo-A, its zebrafish homologues, *rtn4a* and particularly *rtn4b*, are essential for embryonic development and patterning of the nervous system.

## Background

**Figure 1:**
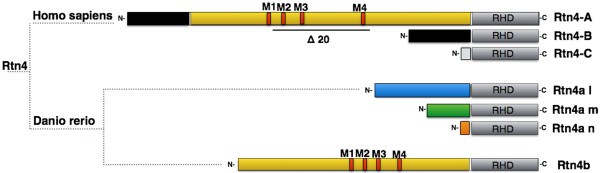
**Schematic representation of the human *RTN4 *gene and its zebrafish paralogues.** All three major isoforms encoded in the *RTN4* gene in humans (RTN4A, RTN4B and RTN4C) possess the reticulon homology domain (RHD), which includes the Nogo66 domain. The N-terminal region of RTN4A contains the Nogo-A-specific domain (yellow) and the neurite growth inhibitory Delta 20 (Δ20) stretch. The diagnostic M1 to M4 motifs are indicated in red. The zebrafish has two *RTN4* paralogues: *rtn4a* and *rtn4b.* Rtn4a is produced in three different isoforms (Rtn4-l (blue), Rtn4-m (green) and Rtn4-n (orange)) with the same C-terminal RHD. Rtn4b also contains the M1 to M4 N-terminal motifs (red) and presents a distinct RHD.

Reticulon 4/Nogo-A is one of the best characterized members of the evolutionarily conserved reticulon (*RTN*) gene family (*RTN1*, *RTN2*, *RTN3* and *RTN4*) [1]. It is also the longest of three *RTN4* gene transcripts A, B and C (Figure 1), as well as a widely known inhibitor of axon regeneration in oligodendrocytes and myelin of the adult mammalian central nervous system (CNS) [2,3]. Growth inhibition is predominantly exerted by two Nogo-A domains, the Delta 20 domain in the N-terminal portion of the protein and the Nogo-66 loop in the C-terminal reticulon homology domain (RHD) [3]. In addition to its activity as an inhibitor of axon growth in the adult CNS, recent studies in mice have uncovered its functional roles in neuronal development and cortical plasticity. For instance, Nogo-A has been demonstrated to be present in migrating neuroblasts and immature neurons in the neural tube, as well as on radially and tangentially migrating neurons of the developing cortex, affecting their motility [4,5]. In other studies, Nogo-A was found to contribute to long-term potentiation (LTP) in the hippocampus, ocular dominance column formation in the visual system, and size control of postsynaptic densities in cerebellar neurons [6-8]. Collectively, these findings suggest that Nogo-A negatively regulates neural plasticity in the mammalian brain [3]. These defects, however, do apparently not interfere with fertility and viability of the Nogo-A-knockout mouse, which shows no striking phenotype at birth [9,10].

Much less is known concerning the role of the RTNs, especially RTN4/Nogo-A, in the neurodevelopment of nonmammalian species. In fish, such analysis is of great interest because axons regenerate successfully in the teleost CNS [11-15] and because neuronal projections in the peripheral nervous system of the embryo seem to develop abnormally when *rtn4a* is downregulated [16].

It has been recognized that zebrafish possess two *rtn4* paralogues, *rtn4a* and *rtn4b* (Figure 1) [17,18]. Both proteins have a conserved RHD, the hallmark of this gene family [1], but very different N-terminal regions [18]. A comparative study revealed that, in contrast to its mammalian counterpart, the Nogo-66 region in the RHD of zebrafish Rtn4, upon binding to either the zebrafish or the mouse Nogo receptor (NgR), promotes neuronal growth [15]. The N terminus of zebrafish Rtn4a bears no resemblance, in sequence or in length, to that of mammalian RTN4, but four short motifs—termed *M1* to *M4* (Figure 1)—were found to be conserved between the N terminus of Rtn4b and the inhibitory Nogo-A-specific Delta 20 domain of mammalian RTN4 [18]. To elucidate the function of the zebrafish Rtn4b N terminus and its M1 to M4 motifs, ongoing studies in our laboratory aim to investigate the expression pattern of Rtn4b in the adult CNS and its potential ability to inhibit axon growth.

Previous work by Brösamle and Halpern [16] addressed the role of zebrafish *rtn4a* using morpholino (MO)-based knockdown strategies and showed that downregulation of the shortest splice form, *rtn4a*-*γ*[17] (hereinafter referred to as *rtn4a-n*), led to misguidance of the posterior lateral line nerve and disorder of cranial nerves in 2- and 3-day-old embryos. Their work further suggested that Nogo–NgR interactions may contribute to axon guidance and to development of the zebrafish peripheral nervous system (PNS) by channeling axons through inhibitory terrain.

Our goal in the present study was to examine the expression and function of *rtn4b* in zebrafish embryos, particularly in light of the similarity between the N-terminal region and that of mammalian Nogo-A/RTN4A (Figure 1). In addition we comparatively analyzed the expression of zebrafish *rtn4a* and its role in embryogenesis. Interestingly, and in contrast to the Nogo-knockout mouse, our results reveal morphological defects in the formation of the spinal cord and brain. In *rtn4b*-knockdown embryos, furthermore, the pectoral fin became absent or reduced and the lower jaw was often lost. Together, the neuronal and non-neuronal defects in *rtn4b* morphants were stronger than those in *rtn4a*, ultimately impairing larval motility and causing death.

## Results

### Expression patterns of *rtn4a* and *rtn4b*

**Figure 2:**
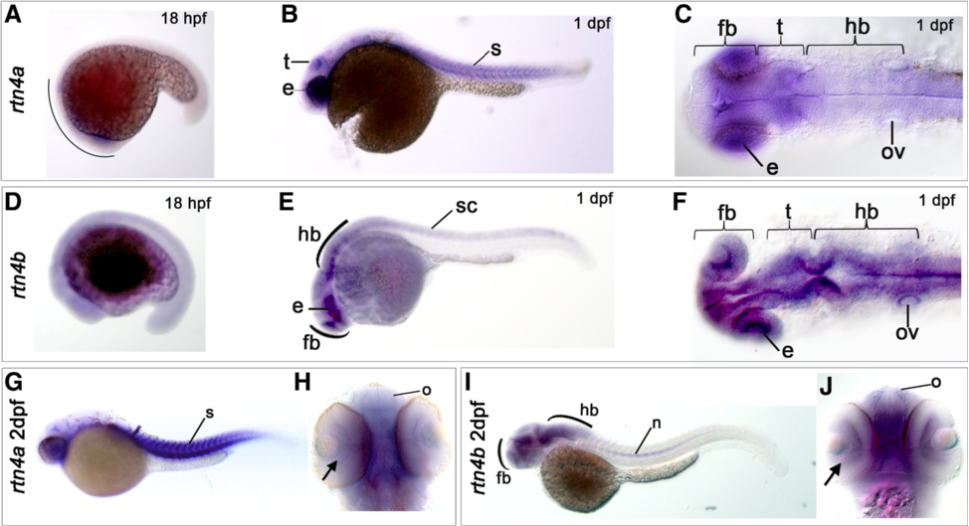
**Expression of *rtn4a *and *rtn4b *genes during zebrafish embryogenesis.** The developmental expression pattern of *rtn4a* and *rtn4b* was examined by whole-mount *in situ* hybridization using gene-specific probes. **(A)** At 18 hours postfertilization (hpf), *rtn4a* is expressed in the anterior part of the embryo. Between 1 day postfertilization (dpf) **(B)** and **(C)** and 2 dpf **(G)** and **(H)**, we detected increased transcription of *rtn4a* in the somites (s) and the eye anlage (e), as well as in the presumptive optic tectum (t). **(D)** At 18 hpf, *rtn4b* transcripts appeared along the trunk of the embryo. **(E)** and **(F)** At 1 dpf, *rtn4b* expression is evident in the posterior spinal cord (sc), the developing forebrain (fb), eye anlagen (e) and midbrain and hindbrain (hb), including the otic vesicle (ov). **(G)** and **(H)** At 2 dpf, *rtn4a* mRNAs are produced in retinal ganglion cells (RGCs) (arrow), the olfactory organ (o) and forebrain (fb), as well as in somites (s). **(I)** and **(J)** At 2 dpf, *rtn4b* is transcribed in the forebrain (fb), including the olfactory organ (o) and RGCs (arrow), and in the midbrain and hindbrain. At this stage, the spinal cord was no longer labeled, but the notochord (n) began to express *rtn4b*. Lateral views are shown, except in (C) and (F) (dorsal views of 1-dpf embryos) and (H) and (J) (ventral views of 2-dpf embryos).

The initial assumption that *rtn4a* was the closest zebrafish homologue of mammalian Nogo-A prompted the characterization of its developmental expression patterns [16]. However, the recent identification of a Delta 20–like region in Rtn4b [18] revealed that this protein is equally or more functionally related to mammalian Nogo-A than to Rtn4a (Figure 1). To gain comparative insight into the embryonic expression domains of both duplicate genes, we performed mRNA *in situ* hybridization with *rtn4a*- and *rtn4b*-specific probes. Although expression of both *rtn4a* and *rtn4b* can be first appreciated in the gastrula 6 hours postfertilization (hpf) [16,19], *rtn4a* transcripts become more abundant and were restricted to the anterior half of the embryo by 18 hpf (Figure 2A). At this stage, *rtn4b* expression, in contrast, was also seen in the posterior part of the embryo (Figure 2D). At 1 day postfertilization (dpf), both *rtn4a* and *rtn4b* mRNAs were highly expressed in cells of the eye anlage and in the midbrain in cells of the presumptive optic tectum (Figure 2C and 2F). In addition, the *rtn4a* signal was detected in somite boundaries at 1 dpf and in skeletal muscle at 2 dpf, as previously reported [16] (Figure 2B and 2G). *Rtn4b* mRNA expression at 1 dpf was absent from somites, and, in contrast to *rtn4a*, its expression was observed in the entire CNS, including forebrain, midbrain and hindbrain, as well as in the spinal cord (Figure 2E and 2F). The strongest *rtn4a* signal at 2 dpf was observed in somites, in the brain region and in retinal ganglion cells (RGCs) (Figure 2G and 2H). Similarly, an *rtn4b* signal at 2 dpf was detected in distinct brain areas, including the olfactory system, telencephalon, optic tectum and hindbrain, as well in as RGCs (Figure 2I and 2J). The spinal cord, however, was no longer labeled at 2 dpf, but the signal became visible in the notochord (Figure 2I), in branchial arches and pectoral fins (not shown). Thus, both genes are strongly expressed in the developing nervous system.

### Morphological defects of *rtn4a* and *rtn4b* knockdown

A previous study reported embryonic roles of *rtn4a* in the PNS up to 4 dpf [16]. Our own expression data are in agreement with these observations (Additional file 1: parts E and F) and additionally suggest the roles of both *rtn4a* and *rtn4b* in the CNS at later stages. Therefore, we examined and compared their developmental functions using the MO-knockdown approach. After running titration and toxicity controls (Additional file 1: part B), MOs targeting the shared 5′ untranslated region (5′UTR) of all three *rtn4a* isoforms, *l*, *m* and *n* (previously known as *α*, *β* and *γ*) [16,17], and against each splice form separately, a second MO against ATG as well as two MOs against *rtn4b* (5′UTR, MO1; ATG, MO2) were separately microinjected, and the embryos were scored for morphological phenotypes (Additional files 1 and 2).

**Figure 3:**
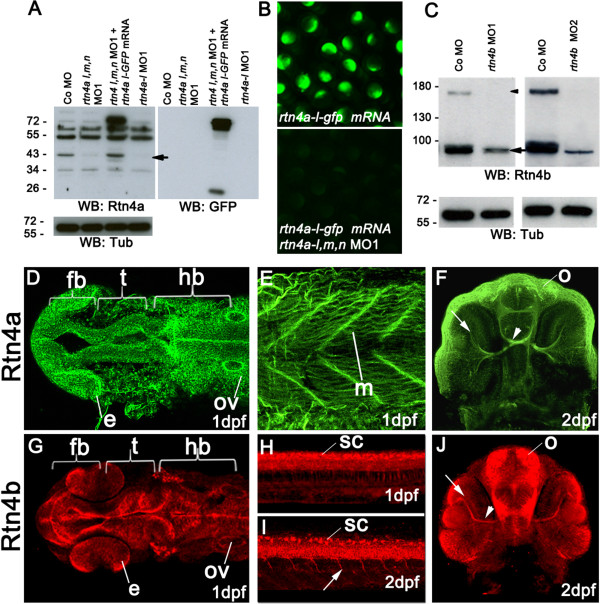
**Rtn4a and Rtn4b protein levels and morpholino knockdown. (A)** Western blot (WB) analysis using the zebrafish α-Rtn4a antibody [15] shows that Rtn4a-l (an approximately 43-kDa band, arrow) is suppressed either by injection of a mixture of morpholinos (MOs) against each *rtn4a* isoform or a MO against the *rtn4a-l* isoform. Embryos expressing an Rtn4a-l-GFP fusion can overcome the Rtn4a MO1 downregulation. GFP antibodies detect the fusion protein at approximately 70 kDa and GFP alone at approximately 26 kDa. α-tubulin (Tub) served as a protein loading control. **(B)** Exogenous Rtn4al-GFP was detected at 6 hpf, but simultaneous coinjection of *rtn4a-l* MO1 abrogated its expression. **(C)** α-Rtn4b antibodies show the downregulation of Rtn4b for both MOs used in the experiments. The 180-kDa band (arrowhead), probably a dimerization band, is entirely reduced in MO-injected embryos, and the 90-kDa band (arrow) shows a strong reduction. **(D)** through **(J)** Rtn4a and Rtn4b immunostainings at various developmental stages. At 1dpf, Rtn4a is expressed in distinct neuronal structures, including the forebrain (fb), the presumptive optic tectum (t) and the hindbrain (hb). Rtn4a is also present in the eye anlage (e), otic vesicle (ov) **(D)**, and in muscle tissue (m) **(E)**. At 2 dpf, Rtn4a is detected in retinal ganglion cells (RGCs) (arrow), the optic nerve (arrowhead) and the olfactory system (o) **(F)**. At 1 dpf, Rtn4b is expressed in the same structures as Rtn4a except the muscle tissue **(G)**. Rtn4b is also detected in the spinal cord (sc) **(H)**. At 2 dpf, the Rtn4b signal is still present in the spinal cord (sc) and can also be seen in growing primary motor neurons (arrow) **(I)**. In the head, Rtn4b is present in RGCs (arrow) and the optic nerve (arrowhead) **(J)**.

In addition to using two MOs against each *rtn4*, we confirmed the specificity of the MOs against *rtn4a* and *rtn4b* by two antibodies: IK964 against the RHD of Rtn4a and K1121 against M1-4 of Rtn4b. In immunoblots of proteins from untreated and MO-injected embryos, the protein-specific bands at 43 kDa (Rtn4al) (Figure 3A) and 90 and 180 kDa (Rtn4b) (Figure 3A and 3C) disappeared or were massively reduced in MO-treated embryos. The blots suggest that Rtn4al is the predominant form because a mixture of all *rtn4a* MOs (MO1) caused the disappearance of only one specific band at 43 kDa. Similarly, when Rtn4a-l- green fluorescent protein (GFP) was overexpressed and detected by anti-GFP, a mixture of MO1 against rtn4a-l, rtn4a-m and *rtn4a-n* led to the loss of GFP fluorescence. That the GFP antibody was able to detect the Rtn4al-GFP fusion protein was confirmed by Western blot analysis (Figure 3B).

We then used the antibodies for detection of Rtn4a and Rtn4b in embryos. In immunostaining experiments, both antibodies labeled neurons and axons in the forebrain, midbrain and hindbrain in 1-dpf embryos (Figure 3D and 3G) and labeled RGCs and their axons, commissures and the olfactory system in the 2-dpf embryo (Figure 3F and 3J), consistent with the distribution of the mRNAs. Also, K1121 against Rtn4b stained cells in the spinal cord at 1 dpf and motor neurons and their axons at 2 dpf (Figure 3H and 3I). IK964 against Rtn4a bound to somites (Figure 3E) and not to the spinal cord or motor neurons. That the antibody staining is specific was shown in embryos injected with MOs against *rtn4a* and *rtn4b*, in which labeling was significantly reduced (Additional file 3). This expression pattern led us to expect morphological defects in MO-injected embryos.

**Figure 4:**
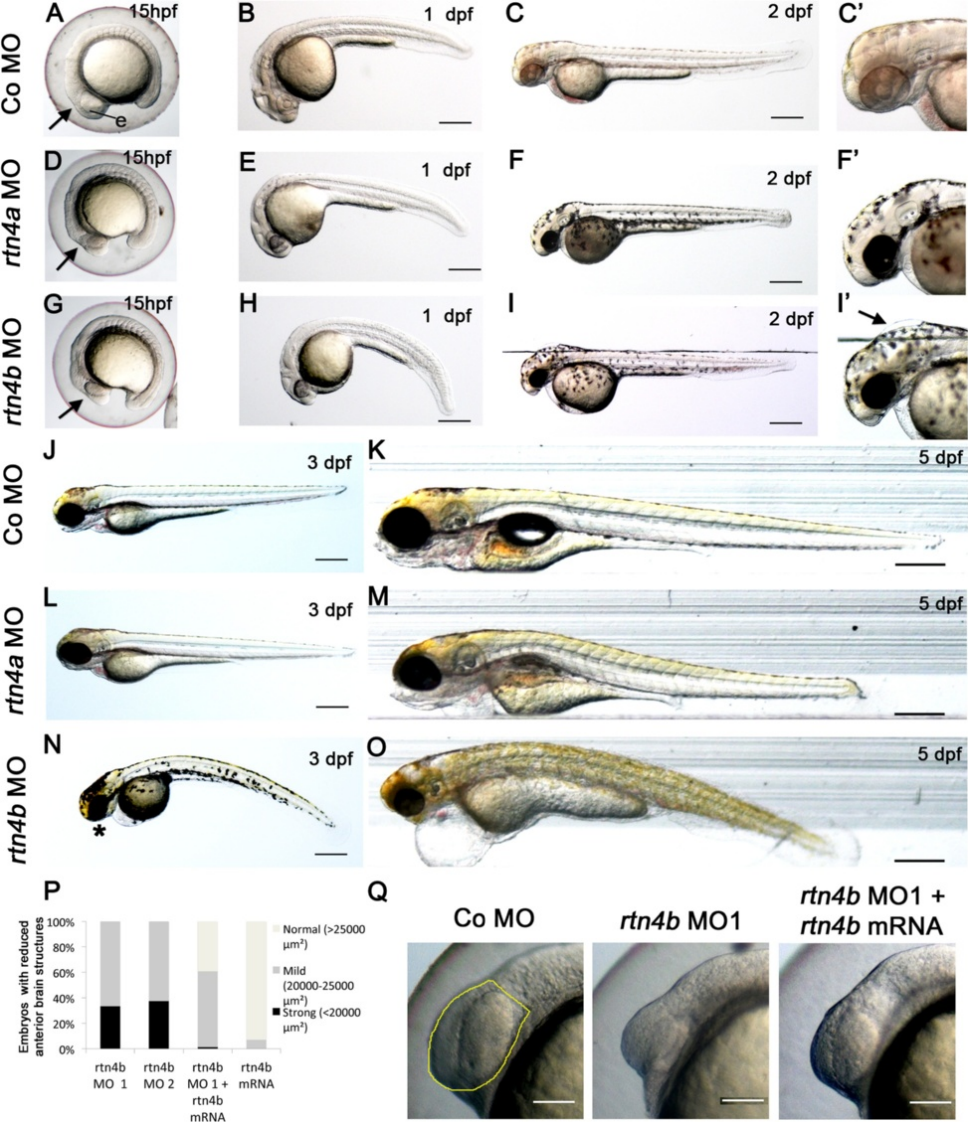
**Morphogenetic defects after morpholino-mediated downregulation of *rtn4a *and *rtn4b*. (A)** At 15 hpf, control embryos showed a differentiated eye anlage (e) and forebrain (arrow). In contrast, the general morphology of *rtn4a-* and *rtn4b*-morpholino (MO)-injected embryos was visibly affected **(D)** to **(G)**. The forebrain was flattened (arrow), and the eye anlage (e) and the head were reduced in size. At 1 dpf, the heads and eyes of embryos injected with *rtn4a* and *rtn4b* MO1 remained reduced. In particular, *rtn4b* morphants exhibited an abnormally curved notochord **(E)** to **(H)**. At 2 dpf, the *rtn4a* morphants still had reduced eyes and forebrains compared to controls (Co) **(F** and **F′** vs. **C** and **C′)**, but no other morphogenetic defects were apparent. *Rtn4b* morphants had even smaller eyes, markedly shortened forebrain/midbrain regions and a deformed fourth ventricle (**I** and **I′**, arrow). At 3 dpf, the eyes and brains of *rtn4a* and *rtn4b* morphants remained smaller **(J, L and N)**. *Rtn4b* morphants developed a thinner, ventrally curved tail; lacked lower jaws (asterisk); and had an inflated heart cavity. At 5 dpf, *rtn4a*, but not *rtn4b*, morphants seemed to regain a nearly normal overall morphology **(K, M and O)**. The reduction in eye size was quantified at 15 hpf in embryos injected with *rtn4b* MO1 and MO2 (targeting the 5′ untranslated region and ATG, respectively) and coinjected with *rtn4b* mRNA **(P)**. In rescue experiments, the *rtn4b* morphant phenotype showed clear improvement **(Q)**. Anterior brain structures selected for measurements are outlined in yellow. Samples studied were *rtn4b* MO1 (*n* = 36), *rtn4b* MO2 (*n* = 64), *rtn4b* MO1 + *rtn4b* mRNA (*n* = 69) and *rtn4b* MO1 (*n* = 85). Scale bars = 100 μm.

Indeed, between 15 and 24 hpf, *rtn4a* morphant embryos microinjected with a mixture of 2 ng of the three *rtn4a* isoforms showed abnormalities most visible in the head region, such as reduced eyes and mild deformations of the forebrain (Figure 4D and 4E). The same defects were also observed in 75% (*rtn4a-l*), 51% (*rtn4a-m*) and 30% (*rtn4a-n*) of the embryos, when 5 ng of MO against each isoform were injected separately (Additional file 1). This shows that Rtn4a-l is apparently the functionally most prominent form, a finding that is consistent with immunoblot results in which IK964 against Rtn4a gave one specific band at 43 kDa, which disappeared after MO knockdown of *rtn4a-l*. When *rtn4a* MO-injected embryos at 2 dpf were stained with the antibody against acetylated tubulin, the pathfinding mistake of the lateral line nerve—first described by Brösamle and Halpern [16] for *rtn4-γ* (that is, *rtn4a-n*)—was one striking defect in the organization of the fiber tracts (Additional file 1: parts E and F). Yet, it was not the sole neurological defect, as described further below in the retina and brain development section.

*Rtn4b* morphants at 15 hpf exhibited similar but stronger defects when injected with 5 ng of MO1. The size of the eyes was reduced and the brains were smaller (Figure 4G, 4P and 4Q). In particular, the forebrain appeared flattened and failed to develop distinct diencephalic and telencephalic regions. At 1 dpf, the *rtn4b* morphants appeared abnormally curved (Figure 4H).

At 2 dpf, the *rtn4a* and *rtn4b* morphants showed increasingly reduced brain sizes (Figures 4F, 4F′, 4I, and 4I′) compared to controls (Figure 4C and 4C′). Moreover, the fourth brain ventricle was expanded in most *rtn4b* morphants at 2 dpf, so that the skin above the hindbrain seemed to have lifted (Figure 4I and 4I′). The cerebellum and posterior hindbrain regions were present. At 3 dpf, the *rtn4b* morphants remained abnormally curved and had lost motility. In addition, they had smaller heads and lacked the lower jaw (Figure 4N). They were impaired in their escape response and reacted to touch with one or two swimming strokes and eventually ceased to move altogether (Additional file 4C). The abnormal forebrain in *rtn4b* morphants caused a shift of the optic tectum into abnormally anterior positions, a phenomenon that became even more pronounced at 5 dpf (Figure 4O and see below, Retina and brain development section). Furthermore, the *rtn4b* morphants showed an increasingly curved tail, an inflated heart cavity and abnormal mouth and jaws, and they died at about 7 dpf. The *rtn4*a morphants, in contrast, were less disturbed in their overall morphology (Figure 4L and 4M). Interestingly, although the motility of *rtn4b* morphants appeared to be reduced relative to control embryos, they always escaped upon touch, and this defect did not result in lethality.

Given the severity of the *rtn4b* morphological knockdown phenotypes, we ran additional tests to rule out unspecific MO effects. To this aim, we performed rescue experiments in which embryos were co-injected with *rtn4b* MO-1 and *rtn4b* mRNA engineered to lack the MO-binding site. *Rtn4b* morphant embryos were evaluated at 14 to 15 hpf and the degree of rescue was assessed as the proportion of embryos exhibiting mild or strong eye and forebrain phenotypes (Figure 4P). In contrast to the 33% strong and 67% mild phenotypes observed among *rtn4b* morphants, only 2% of the rescue embryos had strong phenotypes, 59% had mild phenotypes and 39% looked normal (Figure 4P an 4Q). To rule out that mRNA might induce defects on their own, we examined *rtn4b* mRNA-injected embryos and found that only 7% of them showed mild phenotypes (Figure 4P). The results of these rescue experiments suggest that *rtn4b* MO-1 plays a specific role in brain morphogenesis. This result was supported by experiments with MO2, which caused defects similar to those caused by MO1 in 100% embryos (Figure 4P and Additional file 1: part D).

### Roles of *Rtn4b* in neural development

**Figure 5:**
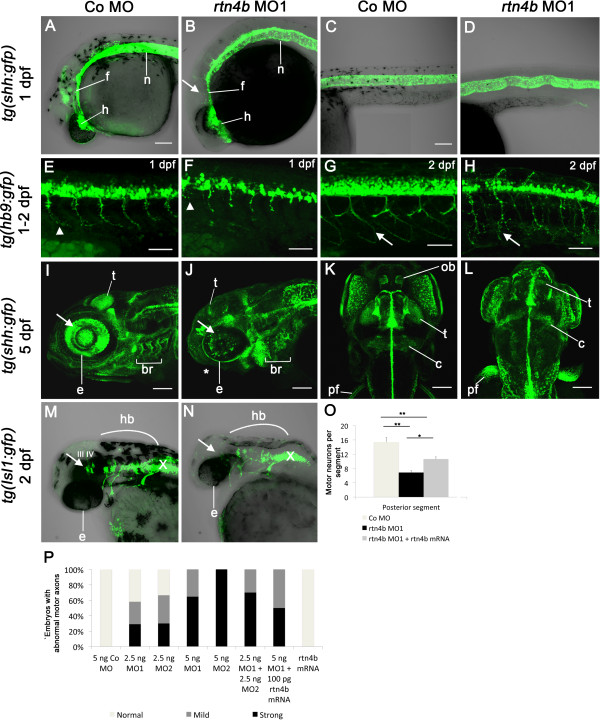
**Development of neural and non-neuronal structures in *rtn4b***-**knockdown, GFP transgenic embryos.** At 1 dpf, *tg*(*Shh*:*GFP), rtn4b* morphant embryos showed deformations in the hypothalamic region (h), dorsal thalamic structures (arrow), anterior floor plate (f) and forebrain **(A)** and **(B)**. Additionally, the notochord (n) is abnormally undulated (compare **(C)** and **(D)**). In addition, 1-dpf *tg(hb9*:*GFP)*, *rtn4b* morphant embryos had fewer cell bodies in the spinal cord and truncated motor axons **(E)** and **(F)** (arrowheads). At 2 dpf, motor axons showed aberrant branching **(G)** and **(H)** (arrows). At 5 dpf, lateral **(J)** and dorsal **(L)** views reveal smaller eyes (e), aberrant retinal ganglion cells (RGCs) (white arrow) and few retinal axons projecting into the tectum (t), which is displaced anteriorly, along with abnormally patterned branchial arches (br) and reduced pectoral fins (pf) **(I)** to **(L)**. At 2 dpf, *tg(Isl1*:*gfp)*, *rtn4b*-MO1 morphants showed severe defects in cranial motor neurons, including lack of pairs III and IV **(M)** and **(N)**. **(O)** Quantification of the number of motor neurons in posterior segments (*n* = 15 to 20) of the spinal cord in control morpholino (Co MO; *n* = 20), *rtn4b*-MO1 (*n* = 25) and rescued (*n* = 20) embryos at 1 dpf. **(P)** Proportion of embryos with abnormal motor axons in embryos injected with Co MO, *rtn4b*-MO, rtn4b-MO2, or coinjected with half concentrations of both MOs, or *rtn4b*-MO1 + *rtn4b*-mRNA, and injected with *rtn4b*-mRNA. hb: hindbrain; e: eye; ob: olfactory bulb; c: cerebellum; X: branchiomeric nerve X. Co MO (*n* = 15), *rtn4b*-MO1 (2.5 ng) (*n* = 30), *rtn4b*-MO2 (2.5 ng) (*n* = 31), *rtn4b*-MO1 (5.0 ng) (*n* = 17), *rtn4b*-MO2 (5.0 ng) (*n* = 11), *rtn4b*-MO1 + *rtn4b*-MO2 (2.5 ng) (*n* = 20), *rtn4b*-MO1 (5.0 ng) + *rtn4b*-mRNA (100 pg) (*n* = 69) and *rtn4b* mRNA (100 pg) (*n* = 85). Scale bar = 100 μm.

To obtain insights into neuronal and non-neuronal defects underlying the severe *rtn4b* morphant phenotype, we analyzed MO-injected *tg*(*shh*:*gfp*)-, *tg*(*hb9*:*gfp*)- and *tg*(*Isl1*:*gfp*)-transgenic embryos. These embryos express GFP in the floor plate, motor neurons, cranial nerves and retinal axons, respectively, and thus allowed us to recognize defects in the relevant structures following MO injections. *Rtn4b* morphants of *tg*(*shh*:*gfp*) fish at 1 dpf showed abnormalities in the forebrain, including the ventral hypothalamic region and the dorsal interthalamic structures (Figure 5A and 5B) [20]. A closer look at the GFP signal in the notochord and floor plate revealed undulations in the rostrocaudal axis instead of a straight row of cells, which we observed in controls (Figure 5C and 5D). Those undulations, although not easily apparent, were also observed in nontransgenic *rtn4b* morphants (Figure 4H). *Rtn4b* morphants of *tg*(*hb9*:*gfp*) zebrafish showed fewer motor neurons at 1 dpf and abnormal motor axon projections at 2 dpf (Figure 5E to 5H) in correlation with the embryo’s inability to move (Additional file 4). The reduction in the number of cells in the spinal cord and the extent of aberrant projections observed in *tg*(*hb9*:*gfp*) embryos injected with *rtn4b*-MO1 were partially rescued after combined injections of *rtn4b*-MO1 and *rtn4b*-mRNA. In posterior regions of the spinal cord (segments 15 to 18) analyzed at 1 dpf, the number of cells was reduced after injection of *rtn4b* MO (approximately 6.87 cells per segment) compared to control MO–injected embryos (approximately 15.37 cells per segment). This reduction was partially rescued upon combined injections of *rtn4b*-MO and *rtn4b*-mRNA (approximately 10.62 cells per segment) (Figure 5O and Additional file 4). Moreover, the proportion of embryos with strong abnormal motor axon projections in *rtn4b* morphants (approximately 75%) was reduced after coinjection of *rtn4b*-MO and *rtn4b*-mRNA (approximately 33%) (Figure 5P). In another group of *tg(hb9:gfp) fish*, we examined whether the embryos at 3 dpf showed an escape response when touched. In the *rtn4b* rescue group, 38% were able to escape, whereas only 5% of embryos escaped in the *rtn4b*-MO group. All embryos were motile in the mRNA-injected group (Additional file 4: part C). The partial rescue by combined *rtn4b*-MO and *rtn4b*-mRNA injections shows that the defects were specific for *rtn4b* MOs (Figure 5O and 5P).

The reduction in number of cells in the spinal cord and the reduced brain size might be a result of increased apoptosis, reduced cell proliferation or both. Indeed, when 1-dpf *rtn4a*- and *rtn4b*-MO-treated embryos (MO1 for *rtn4a* and *rtn4b*) were exposed to acridine orange (apoptotic cells), significant increases in labeled cells was recorded in the midbrain and hindbrain (Additional file 5).

In embryos exposed to bromodeoxyuridine (BrdU) at 1 dpf and analyzed 1 hour later, BrdU and 4′,6-diamidino-2-phenylindole (DAPI) labeling showed a reduction in the total number of cells in *rtn4a*-MO-injected embryos, and an even greater reduction in *rtn4b*-MO-injected embryos (Additional file 6). Interestingly, the ratio of BrdU-positive cells was identical in controls and following *rtn4a*-MO or *rtn4b*-MO injections, indicating that the number of cells entering the S-phase was not affected (Additional file 6: parts H and P).

In a pulse-chase experiment in which embryos were given BrdU at 1 dpf and analyzed 2 days later, it was apparent that the BrdU labeling was retained in most cells of the midbrain and hindbrain of 3-dpf *rtn4b*-MO-treated embryos (see Additional file 6 for BrdU labeling in the tectum and a related quantification in the hindbrain), suggesting that cell proliferation was impaired in these structures. This observation is consistent with the massive reduction of brain size in these embryos (Figure 4P). On the other hand, *rtn4a*-MO-treated embryos showed a reduction in BrdU labeling similar to that in control MO-injected embryos (Additional file 6: parts E and N). Interestingly, although their brains were smaller than those of control embryos at 1, 3 and 5 dpf (Figure 4), this effect was not as severe as it was in *rtn4b* morphants. Therefore, the rate of cell division in *rtn4a* morphants was likely affected only transiently or partially.

At 5 dpf in *rtn4b* morphants of the *tg*(*shh*:*gfp*) line (Figure 5I to 5L), the absence of the lower jaw and of the five branchial arches, which will form the gills, became evident. Moreover, the *rtn4b* morphants had problems with visual system development. RGCs and their axonal projection to the optic tectum were underdeveloped, and the optic tectum shifted anteriorly (Figure 5I to 5L and next section). Moreover, the pectoral fins were abnormal or missing. In addition, the *rtn4b* morphants of *tg(Isl1:gfp)* embryos showed that the neurons of cranial nerves III and IV failed to differentiate (Figure 5M and 5N). Thus, *rtn4b* morphants had severe brain-patterning defects and skeletal malformations.

### Retina and brain development

**Figure 6:**
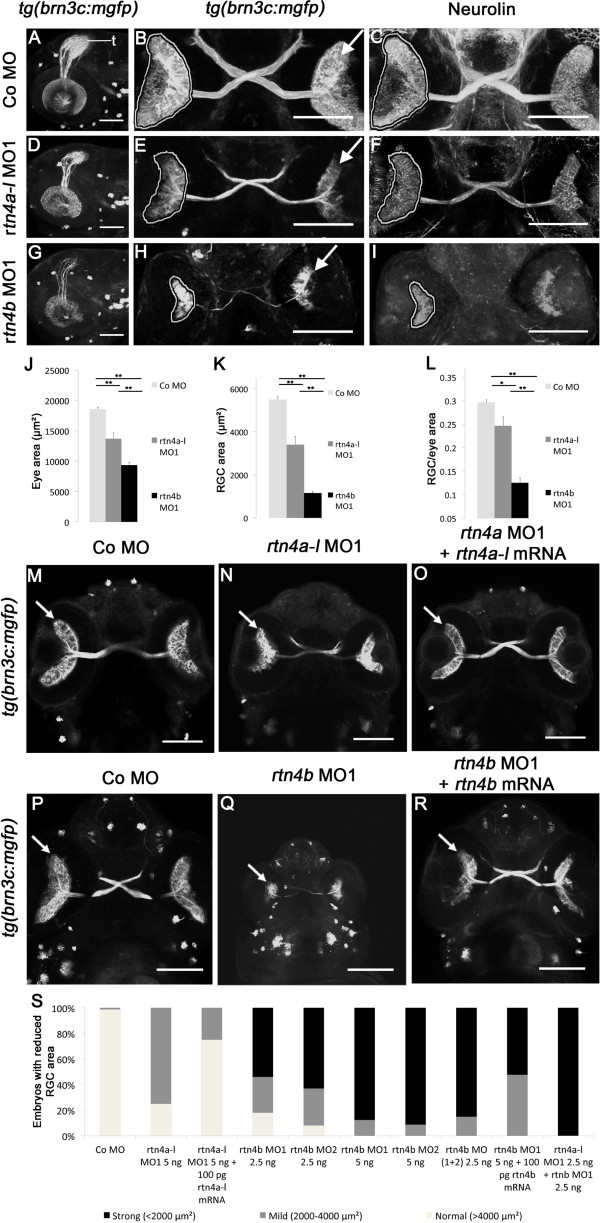
**Retinal ganglion cell development in *rtn4a*- and *rtn4b***-**knockdown, GFP–transgenic embryos. (A)** through **(I)***tg(brn3c:mGFP)* embryos at 3 dpf exhibit labeled retinal ganglion cells (RGCs) in the eye (arrow) and RGC axons in the optic nerve and tract as well as in the optic tectum (t). **(A)**, **(D)** and **(G)** Lateral views of the left eye and optic tectum (anterior to the left). **(B)**, **(C)**, **(E)**, **(F)**, **(H)** and **(I)** Ventral views (anterior to the top). **(A)** to **(C)** In control morpholino (Co MO)–injected embryos, RGC axons reached the optic tectum (t) and innervated the neuropil **(A)**. The ventral view shows the RGCs in both eyes (arrow) and the RGC axons in the optic nerve, the chiasm and the optic tract **(B)**. **(C)**, **(F)** and **(I)** RGCs and their axons were costained with antibodies against neurolin. RGCs from both morphants, *rtn4a***(D)** to **(F)** and, to a greater extent, *rtn4b***(G)** to **(I)**, cover smaller areas of the retina (outlined) and their axons show aberrant pathways. **(J)** to **(L)** Quantification of eye size, the area covered by RGCs and their ratio. **(M)** to **(O)** and **(P)** to **(R)** Rescue of the *rtn4a* and *rtn4b* RGC phenotypes. Arrow points to RGCs. **(S)** Quantification at 3 dpf showing the reduction in the area covered by RGCs after injection of *rtn4a*- and *rtn4b*-MO1 (separately and combined) and *rtn4b*-MO1 and *rtn4b*-mRNA, as indicated under each bar. Control-MO (*n* = 28), *rtn4a-l*-MO1 (5.0 ng) (*n* = 16), *rtn4a-l*-MO1 (2.5 ng) + *rtn4a-l*-mRNA (100 pg) (*n* = 32), *rtn4b*-MO1 (2.5 ng) (*n* = 22), *rtn4b*-MO2 (2.5 ng) (*n* = 24), *rtn4b*-MO1 (5.0 ng) (*n* = 19), *rtn4b*-MO2 (5.0 ng) (*n* = 24), *rtn4b*-MO1 and *rtn4b*-MO2 (2.5 ng) (*n* = 27), *rtn4b*-MO (5.0 ng) and *rtn4b*-mRNA (100 pg) (*n* = 23) and *rtn4a-l*-MO1 (2.5 ng) and *rtn4b*-MO1 (2.5 ng) (*n* = 27). Scale bar = 100 μm.

Besides the clear differences between the *rtn4a* and *rtn4b* expression patterns and knockdown phenotypes, some similarities were also evident. For instance, both transcripts appeared to be upregulated in the optic tectum and retina, and both morphants exhibited related morphological defects, such as smaller brains and eyes. To better characterize neural roles of *rtn4* paralogues, we analyzed their function in the GFP-labeled retinotectal system of *tg(brn3c:mgfp)* zebrafish. Injection of *rtn4a-l* MO1, but more severely *rtn4b* MO1, resulted in a reduction in the size of the eyes of 27% and 50%, respectively, and a reduction in the area covered by RGCs of 39% and 80%, respectively (Figure 6J to 6L). *Rtn4b* MO1 and *rtn4b* MO2 at half concentrations enhanced each other and led to 85% severely and 15% mildly reduced RGC areas (Figure 6S). *Rtn4b* MO2 caused a strong reduction in the area occupied by RGCs in 91% of the embryos (and 9% mild). Moreover, *rtn4a-l* MO1 combined with rtn4b MO1 at half concentration acted synergistically, causing this effect in 100% of the embryos.

In 3-dpf *rtn4a* morphants, the area of the retina occupied by RGCs was reduced and extended markedly fewer axons into the optic nerve and tectum (*rtn4a*-MO1; Figure 6D and 6E) than in control embryos (Figure 6A and 6B). The *rtn4b* morphant was more severely affected, as the area occupied by GFP-labeled RGCs was massively reduced and the few axons formed rudimentary optic nerves (*rtn4b*-MO1; Figure 6G and 6H). To rule out the possibility that axons were present in both morphants but failed to express GFP, we performed immunostaining with antibodies against the cell adhesion molecule neurolin. These immunostained images show that the RGCs and axons were indeed reduced (rather than being invisible as a result of the absence of GFP expression) (Figure 6C, 6F and 6I). The specific roles of *rtn4a* and *rtn4b* in retinotectal development were also confirmed by rescue experiments in which mRNAs of the *rtn4a* (isoform l) and *rtn4b*, respectively, were coinjected along with the corresponding MOs. Upon coinjection of 100 pg of *rtn4a-l*-mRNA, morphants with mild phenotypes were reduced from 75% to 25% (Figure 6M to 6O and 6S). The concentration-dependency of the mRNA rescue experiment strongly suggests that the phenotypes observed were specifically caused by *rtn4a* downregulation. Likewise, the *rtn4b* (MO1) strong phenotype was partially rescued by mRNA coinjection. Morphants with strong phenotypes were reduced from 87% to 52% (Figure 6P to 6R and 6S). The remaining 48%, however, remained mildly affected. Besides supporting the specificity of the MO effects on RGC differentiation and RGC axon growth, these experiments also confirm the strong effect of *rtn4b* downregulation on retinotectal development.

**Figure 7:**
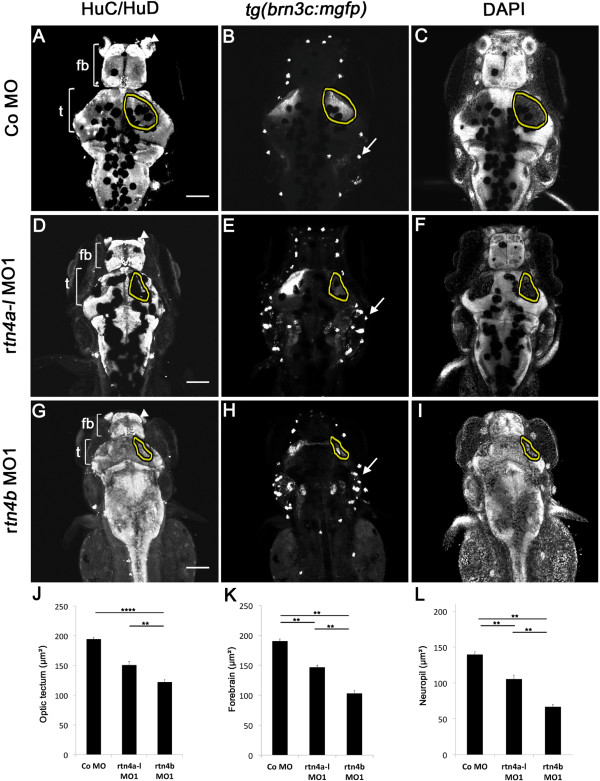
**Brain differentiation and retinotectal projections in *rtn4a*- and *rtn4b***-**knockdown, GFP–transgenic embryos.** In *rtn4a*-morpholino (MO)- and *rtn4b*-MO-injected embryos, brains are smaller both anteroposteriorly as well as laterally. **(A)** Extent of the tectum (t) and the forebrain (fb) marked by white brackets in the HuC/HuD labeled brains of control embryos. **(D)** and **(G)** Knockdown of *rtn4a*, but more severely of *rtn4b*, leads to a reduction in the size of the tectum and the forebrain. In *rtn4b* morphants, the olfactory placodes (arrowhead) are not clearly identifiable and the tectum is localized in abnormally anterior positions. **(B)** In control embryos, the GFP–labeled retinal ganglion cell (RGC) axons cover the tectal neuropil (outlined). **(E)** and **(H)** The RGC axons in the tectum cover a smaller area in both morphants, but more severely so in the *rtn4b* morphants than in their *rtn4a* counterparts. GFP is also expressed in neuromasts (arrows), which are aberrantly positioned in the morphants. **(C)**, **(F)** and **(I)** 4′,6-diamidino-2-phenylindole (DAPI) staining of the brain shows the area of the neuropil (outlined), which is reduced after downregulation of *rtn4a* and *rtn4b*. **(J)**, **(K)** and **(L)** Quantification of the length of the optic tectum, forebrain and tectal neuropil in control and *rtn4a*-MO- and *rtn4b*-MO-injected embryos. **(A)** to **(I)** Dorsal views of the brain of *tg(brn3c:mGFP)* embryos at 5 dpf. Control MO (5.0 ng) (n = 10), *rtn4a-l*-MO1 (5.0 ng) (n = 10) and *rtn4b*-MO1 (5.0 ng) (*n* = 10). Scale bar = 100 μm.

At 5 dpf, most *rtn4a* morphants of the *tg(brn3c:mgfp)* embryos still had smaller retinotectal projections (Figure 7D and 7E) but the reduction in size was more severe in *rtn4b* morphants (Figure 7G and 7H). The tectum resides in positions far too anteriorly, as mentioned above (Figure 7G). Brains exposed to the neuronal marker HuC/HuD and the nuclei marker DAPI, which stain all cells and spare the tectal neuropil, highlight how small the neuropil had become in the morphants compared to controls (Figure 7A, 7C, 7D, 7F, 7G and 7I). A comparison with the controls illustrates that the tectum, the forebrain and the neuropil in the *rtn4b* morphants were reduced to 62%, 54% and 48%, respectively, of their original size (Figure 7G and 7J to 7L) and to 78%, 77% and 76%, respectively, in the *rtn4a* morphants (Figure 7D and 7J to 7L). Again, this analysis underscores the abnormal brain patterning in *rtn4b* morphants, which is more severe than in the *rtn4a* morphants. In addition to RGCs, *tg(brnc3:mgfp)* also expresses GFP in neuromasts. The number of neuromasts and their position was also abnormal in the morphants (Figure 7B, 7E and 7H).

In sum, downregulation of both *rtn4a* and *rtn4b* produced clear abnormalities in the development of the CNS and the PNS, whereby the strongest effects were observed in *rtn4b* morphants. In these embryos, specific neural structures such as the retina, the optic tectum and the forebrain, as well as neuromasts, cranial ganglia and spinal cord, were markedly reduced. Loss of *rtn4b* also led to reduced and disorganized axon projections from RGCs, cranial ganglia and motor neurons, and ultimately to larval death. Outside the nervous system, *rtn4b* morphants suffered from the loss of lower jaws, shorter or absent pectoral fins and notochord abnormalities in correlation with the increasingly curved tail. In *rtn4a* morphants, the retinotectal defects were qualitatively similar to those in *rtn4b* morphants, but overall were less severe and apparently reversible with time. The cranial nerve and lateral line misprojections of *rtn4a* morphants reported by Brösamle and Halpern [16], together with early forebrain defects, seemed not to be lethal, at least not in the first weeks of life.

## Discussion

Although knockout of RTN4/Nogo-A in mice produces viable animals without obvious morphological defects in the brain [9,10], our study results show clear neurodevelopmental defects upon downregulation of both zebrafish RTN4 homologues. In particular, zebrafish *rtn4b* plays essential roles in the early development of the nervous system and in the morphogenesis of the notochord and jaws. In the nervous system, the most striking effects of *rtn4b* downregulation were reduced number of motor neurons and abnormal motor axon pathfinding, reduced eye and brain size, abnormal retinotectal projection, fewer and mislocalized neuromasts and axon pathfinding errors of cranial nerves and nerves of the anterior and posterior lateral lines. The severity and lethality of these defects indicate that, unlike zebrafish *rtn4a* and mammalian *RTN4*, *rtn4b* is vital for zebrafish embryonic development. Our MO data further show that zebrafish *rtn4a* also contributes to CNS and PNS development, although to a lesser degree than *rtn4b*. This synergy between the neurodevelopmental roles of *rtn4a* and *rtn4b* is consistent with their expression in partially overlapping domains of the brain. Differential expression was restricted mainly to posterior regions of the embryo, where *rtn4a* was detected in lateral line ganglia and somites, and *rtn4b* was detected in the spinal cord. Unlike *rtn4b*-knockdown embryos, *rtn4a* morphants, although less motile relative to controls, always escaped upon touch, survived for weeks and seemingly recovered some of the earlier abnormalities.

Among the most striking abnormalities shared between the *rtn4a* and *rtn4b* morphants were their strongly reduced and malformed retinotectal projections. The tecta appeared to have shifted into abnormally anterior positions, possibly as a consequence of the reduced forebrain size, and received a small number of RGC axons from the few remaining RGCs. This observation suggests that *rtn4a* and *rtn4b* are involved in neuronal differentiation and/or the maintenance of normal cell numbers in specific areas of the nervous system and therefore may be required for cell proliferation, cell survival and pattern formation in specific subdivisions of the CNS and PNS. Our previous bioinformatic analyses strongly suggest that the Rtn4b N-terminal domain is directly homologous to the corresponding Delta 20-containing region of mammalian Nogo-A [18]. However, downregulation of RTN4/Nogo-A in mammals does not obviously impair cell proliferation, neuronal differentiation or brain patterning in the early embryo [9,10]. Interestingly, the absence of Nogo-A/RTN4 and the use of Nogo-blocking antibodies have been shown to increase the rate of radial migration in hippocampal, cortical and cerebellar neuronal progenitors [4,5]; to impair synaptic potentiation in the hippocampus [7]; and to affect the size of cerebellar postsynaptic densities [8]. This is consistent with a significant role of mammalian RTN4/Nogo-A as a negative regulator of cortical plasticity in developmentally older embryos, in contrast to our observations in fish, in which defects appeared much earlier in development.

Notably, the authors of a recent report uncovered distinct neurodevelopmental roles for the mouse Delta 20 and Nogo-66 regions [21]. In that study, proliferation of neural stem cells in the adult subventricular zone was found to be modulated by Nogo-66/NgR1 interactions, whereas the migration of neuroblasts to the olfactory bulb was controlled by binding of Delta 20 to a receptor complex distinct from NgR1. Conservation of related activities in zebrafish *rtn4* paralogues may be relevant to our finding of reduced neural structures in morphant embryos. Our data further suggest that the function of Nogo genes evolved independently in fish and mammals, with an early developmental role becoming more predominant in the former and a later function in cortical development and plasticity in the latter. We presently do not know whether this difference might involve the action of the Delta 20-like region of zebrafish *rtn4b* as an inhibitor or a repulsive cue during cell–cell interaction and axon growth. Remarkably, although zebrafish and mammalian Nogo-66 are almost 70% homologous, the interaction of zebrafish Nogo-66 (of Rtn4a) and NgR does not lead to inhibition of neurite growth in fish or mammalian neurons but to axon growth [15]. Hence, it remains to be clarified how the inhibitory potential of Rtn4a and Rtn4b may have evolved differentially in fish and mammals.

The reduced brain size and aberrant axonal pathways seen upon *rtn4a* and *rtn4b* knockdown could theoretically result from insufficient signaling through NgR (and coreceptors), but this phenomenon remains to be analyzed. Interestingly, although NgR1 is expressed in the embryonic zebrafish brain [22], its downregulation is known to cause pathfinding errors only in PNS axons [16]. It is presently unknown whether CNS fiber tracts are affected by NgR knockdown or whether the Nogo-66 domain of Rtn4b binds to NgR. Similarly, whether the Delta 20-like region of Rtn4b binds to a receptor complex resembling the mammalian amino-Nogo-A receptor complex needs to be clarified. Yet, some conservation between the interactions of Delta 20 and Nogo-A receptor in fish and mammals is expected, based on their sequence similarity [18] and on the fact that fish axons recognize mouse Nogo-A Delta 20 [15,23].

Most assumptions about the function of RTN4A/Nogo-A are based on its cell surface expression. However, it should be noted that this protein is by far more abundant in the endoplasmic reticulum (ER), where it has been proposed to play a role in structuring the membranous network [24-26]. It has also been reported to be upregulated in mammalian RGCs after optic nerve transection, but no concrete function has been associated with this phenomenon [27]. The subcellular localization of the zebrafish Rtn4 homologues has not been examined in detail, but Nogo-66 has been shown to reside in and on glial cells in the adult regenerating optic nerve [19], as was also demonstrated for mammalian Nogo-66 and the Nogo-A-specific region [3].

## Conclusions

Both *rtn4a* and *rtn4b* functions are required during early zebrafish development, as their downregulation has more dramatic effects in brain patterning than *rtn4* knockout in mammals. This is somewhat surprising, given the fact that zebrafish *rtn4b* and mouse *Rtn4* are expressed in the neural tube and at comparably early stages of brain development [3]. Duplication of genes is often accompanied by the acquisition of a new function of one or both duplicates, as judged by their temporal or spatial expression patterns [28]. Even though *rtn4a* and *rtn4b* differ partially in their expression domains, in that *rtn4a* is, for instance, expressed in somites and *rtn4b* is expressed in the spinal cord and notochord, their expression patterns large overlap. Moreover, their downregulation causes similar abnormalities in forebrain and midbrain morphology as well as in rudimentary and aberrant retinotectal projections. The most significant differences between *rtn4b* and *rtn4a* morphants was the immobility and lethality of *rtn4b* at 5 dpf and the significant recovery and nearly normal outer appearance observed at this time in *rtn4a*. This difference might be causally related to an important function of the zebrafish Rtn4b N terminus, which, like mouse Nogo-A but unlike zebrafish Rtn4a, contains conserved M1 to M4 protein motifs [18]. Nogo-A Delta 20 has been identified as a CNS myelin-associated inhibitor of axon growth and regeneration in the adult mammalian CNS and regulator of plasticity [3]. In this context, it will be interesting to explore the biological properties of the zebrafish Rtn4b Delta 20-like domain and its expression in adult fish, particularly because zebrafish successfully regenerate RGC axons in the optic nerve and fiber tracts in the spinal cord [14,29].

## Methods

Zebrafish (*Danio rerio*) were maintained at 28°C under a 14-hour light, 10-hour dark cycle [30]. Developmental stages are indicated based on those described by Kimmel *et al*. [31] and in hours and days postfertilization (hpf and dpf, respectively). Some embryos were raised in fish water containing 0.003% 1-phenyl 2-thiourea to prevent pigmentation [32]. A zebrafish reporter line expressing GFP under the control of the *sonic hedgehog* gene promoter *tg(shh:gfp)* was obtained from Max-Planck-Institute Developmental Biology (Tübingen, Germany). *tg(hb9:gfp)*-transgenic zebrafish expressing GFP in motor axons were provided by D Meyer (University of Innsbruck, Austria). *tg(Isl1:gfp)* zebrafish expressing GFP in cranial motor neurons were provided by S Higashijima (Okazaki Institute for Integrative Bioscience, Higashiyama, Japan) and *tg(brn3c:mgfp)* zebrafish expressing membrane-targeted GFP in retinal axons were provided by H Baier (University of California, San Francisco, USA).

### Whole-mount *in situ* hybridization

Whole-mount *in situ* hybridization was performed as described previously [33]. We cloned 1.3 kb of *rtn4a-l*, 1 kb of *rtn4a-m* and 0.9 kb of *rtn4a-n* (including the full open reading frames (ORFs) and 393 bp from the 3′UTR and 1.5 kb from the *rtn4b* N terminus, including the M1 to M4 motifs) in pCRII TOPO (Invitrogen, Carlsbad, CA, USA) and used them as templates for the synthesis of two independent RNA *in situ* hybridization probes with the DIG RNA Labeling Kit (Roche Applied Science, Penzberg, Germany). Transcription patterns were visualized using an Axioplan 2 compound microscope (Carl Zeiss Microscopy, Oberkochen, Germany) using Nomarski (differential interference contrast) optics, photographed with a Zeiss Color Axiocam and further processed using Adobe Photoshop 12.0 software (Adobe Systems, San Jose, CA, USA).

### Cloning full-length *rtn4a* and *rtn4b* cDNAs

The *rtn4a* full coding sequence was amplified by RT-PCR from 1-dpf zebrafish embryo total RNA with the following primers: forward *rtn4a*-fw 5′-atgcagccgcaggagtacat-3′ and reverse *rtn4a*-rv 5′-ggctgccgggtcacgact-3′. The *rtn4b* cDNA was amplified with forward primer *rtn4b*-fw 5′-gtcctgagctgcgctatttc-3′ and reverse primer *rtn4b*-rv 5′-gttatttagtaggcagcggtgtg-3′ by RT-PCR from total RNA extracted from adult zebrafish optic nerve. First-strand cDNA was synthesized under standard conditions with the SuperScript First-Strand Synthesis System (Invitrogen) using an oligo(dT) primer. All of the above-mentioned PCR experiments were done with Phusion High-Fidelity DNA Polymerase (Finnzymes/Thermo Fisher Scientific, Espoo, Finland). Full-length cDNAs were cloned into a PCR2.1 TOPO vector (Invitrogen) and sequenced.

### Morpholino knockdowns and mRNA rescue

The following MOs were purchased from Gene Tools (Philomath, OR, USA) and designed to target independent sequences at the 5′ UTRs and the start codon of the zebrafish *rtn4a* and *rtn4b*, including known splice variants based on the following sequence data obtained from the GenBank database (see Additional file 2):

*rtn4a-l*, 5′-taaagtaacttcaagatgcgccgga-3′ (position on mRNA -55/-30) and 5′-tcgtggagcttatttgatcatccat-3′ (position on mRNA 1/25) [GenBank:AY555039.1]; *rtn4a-m*, 5′-cgtgcatcggtcatatatccagtca-3′ (position on mRNA -18/+7) and 5′-ttatctgaattggcgtgcatcggtc-3′ (position on mRNA -5/+20) [GenBank:AY555042.1]; *rtn4a-n*, 5′-ctcgctcattctgcgatcagacagcc-3′ (position on mRNA -25/0) and 5′-gctccaccacttgtttggaatccat-3′ (position on mRNA 1/25) [GenBank:AY555043.1]; *rtn4b*, 5′-ccactgcgggagaactcagaacagc-3′ (position on mRNA -81/-57, for better distinction, *rtn4b*-MO-1) and 5′-gctcgttctgtgtcctccatcggga-3′ (position on mRNA -5/+20, *rtn4b*-MO-2) [RefSeq:NM_001040335.1]; control, 5′-aacgaacgaacgaacgaacgaacgc-3′.

In addition to ATG-targeting MOs, as described by Brösamle and Halpern [16], we used MOs directed against 5′UTR sequences of the *rtn4a* splice variants.

All microinjections were performed at early cleavage stages (one- to four-cell stage) using a manual micromanipulator (Narishige, Tokyo, Japan) coupled to a Transjector 5246 (Eppendorf, Hamburg, Germany) under a Stemi 2000 stereomicroscope (Carl Zeiss Microscopy). After running specificity and dose-dependency controls, MOs were injected at a concentration of 0.5 or 1.0 ng/nl in 13 Danieau buffer (58 mM NaCl, 0.7 mM KCl, 0.4 mM MgSO_4_, 0.6 mM Ca(NO_3_)_2_, 5.0 mM 2-[4-(2-hydroxyethyl)piperazin-1-yl]ethanesulfonic acid [pH 7.6]) and 0.125% Phenol Red (Sigma-Aldrich, St Louis, MO, USA).

For MO rescue experiments, *rtn4a-l* was cloned in frame with GFP into the EcoRI/ApaI restriction sites of pGFP-N1. *RTNa-l-gfp*, *rtn4a-l* and *rtn4b* ORF cDNAs were subcloned into the EcoRI/XbaI (*rtn4al*-gfp), EcoRI/XbaI (*rtn4a-l*) or EcoRI/StuI (*rtn4b*) restriction sites of pCS2+ (provided by Z Varga, University of Oregon, Eugene, OR, USA) and transcribed *in vitro* using the mMESSAGE mMACHINE SP6 kit (Ambion, Austin, TX, USA).

For mRNA synthesis, DNA templates were linearized with BssHII. After synthesis, template DNA was removed by DNaseI digestion of the *rtn4a-l* and *rtn4b* mRNAs. *rtn4a-l* or *rtn4b* MO at 1.0 ng/nl in 13 Danieau buffer were coinjected with capped mRNAs at 20 or 100 pg/nl at a 1:1 ratio in 0.05 M KCl and 0.125% Phenol Red. For overexpression experiments, mRNAs were microinjected at 100 pg/nl. At least 200 embryos per experiment were microinjected (5-nl injection volume) and kept in E3 medium (5 mM NaCl, 0.17 mM KCl, 0.33 mM CaCl_2_ and 0.33 mM MgSO_4_) at 28°C. Quantification of phenotypes was carried out on 200 embryos per experiment, from among which a smaller number were selected for detailed analysis. Images were acquired using a SteREO Lumar.V12, Axioplan 2 or confocal laser scanning microscope LSM 710 (Carl Zeiss Microscopy). Images were further processed using Adobe Photoshop 12.0 software.

### Immunohistochemistry

Anesthetized embryos (6 to 24 hpf) were fixed in 4% paraformaldehyde (PFA) in phosphate-buffered saline (PBS) for 2 hours at room temperature (RT) or overnight at 4°C. Embryos/larvae older than 48 hpf were fixed in PFA for 30 minutes at RT, washed in PBS-Tween 20 (PBST) and permeabilized in acetone for 7 minutes at -20°C. The following antibodies and concentrations were used for whole-mount immunohistochemistry: polyclonal anti-neurolin, 1:500 [34]; monoclonal antiacetylated tubulin, 1:1,000 (Sigma-Aldrich) and the monoclonal anti-HuC/HuD neuronal protein (16A11) 1:1,000 (Molecular Probes, Sunnyvale, CA, USA). For staining with the polyclonal Rtn4a antibody (IK964, which was generated in our laboratory) diluted 1:250 [15], PFA fixation was not used. Instead, embryos were incubated on ice in 50% methanol in PBS, pH 7.4 (2 minutes), 100% MeOH (5 minutes) and 50% MeOH in PBS (2 minutes). To generate a polyclonal antibody against zebrafish Rtn4b, the *rtn4b-M1-M4* region [18] was amplified by PCR from a pCR2.1 TOPO vector containing the *rtn4b* ORF. Forward *rtn4b*-M1-fw 5′-GG*GAATTC*TAGCCCGTCTCCAGACCTGCTCCAGGA-3′ and reverse *rtn4b*-M4-rv 5′-GG*GTCGAC*CTA-CTGCAGACCCTGGAGCAGCTCTGCC-3′ primers containing EcoRI and SalI restriction enzyme sites were designed to amplify 490 bp, including the M1 to M4 motifs. The PCR product was digested with EcoRI and SalI and cloned in frame into the pGEX-4 T-3 glutathione *S*-transferase expression vector (GE Healthcare Life Sciences, Freiburg, Germany) after the thrombin cleavage site. The recombinant protein was used to immunize a rabbit to produce the polyclonal antibody K1121. The immunopurified Rtn4b antibody was used at a dilution of 1:500.

Nuclei were counterstained with 100 ng/ml DAPI, together with the secondary antibody, for 30 minutes at RT. The secondary antibodies were cross-purified with fluorophore-conjugated goat anti-rabbit and cyanine 3 or Alexa Fluor 488–coupled anti-mouse antibodies in which specimens were incubated overnight at 4°C. For analysis of Rtn4 expression levels, embryos were dechorionated, deyolked, lysed and analyzed by Western blotting. Blots were exposed to polyclonal Rtn4a antibody (IK964; diluted 1:10,000) and polyclonal Rtn4b antibody (K1121; diluted 1:1,000) and to a monoclonal antibody against GFP (diluted 1:2,000 to detect Rtn4al-GFP; Roche Applied Science).

### Bromodeoxyuridine labeling

To label cells in the S-phase, embryos were immersed in 10 mM BrdU (Sigma-Aldrich) in 1% dimethyl sulfoxide in E3 medium. Embryos were incubated for 1 hour at 28°C and washed in E3 medium (three timer for 5 minutes), fixed in 4% PFA overnight at 4°C and dehydrated in methanol at -20°C. After gradual rehydration, embryos were permeabilized with proteinase K (10 μg/ml) followed by postfixation with 4% PFA, washed with PBST, blocked with 10% normal goat serum in PBST for at least 2 hours at room temperature and incubated with mouse anti-BrdU-fluorescein isothiocyanate antibody (1:200; Sigma-Aldrich) in 4% blocking solution overnight at 4°C.

### Acridine orange staining

To get an impression of the extent of apoptosis, 1-dpf live embryos were incubated in 2 μg/ml acridine orange (Sigma-Aldrich) for 30 minutes, followed by three rinses in E3 medium. Embryos were anesthetized in 0.016% Tricaine methanesulfonate (MS-222; Sigma-Aldrich) and photographed (Zeiss Lumar.V12 stereomicroscope).

### Motility tests

To evaluate the escape response, 3-dpf embryos were touched with the tip of a fine needle twice at the dorsal tip of the tail. Embryos that did not react were classified as nonmotile. Three groups of at least 50 embryos were tested in each experiment.

### Quantifications

To quantify total cell numbers and axon branching of motor neurons in *tg(hb9:gfp)*, control and *rtn4b*-MO1-injected embryos, six representative specimens from each group were fixed at 1 and 2 dpf, respectively, and their trunk regions were scanned by confocal microcopy. All fluorescent cells (trunk segments 15 to 18) and axonal projections (trunk segments 5 to 8 and 15 to 18) were counted in z-stack confocal reconstructions. Embryos exhibiting aberrant branching and mistakes in pathfinding of their motor axons were classified as mild, and those which in addition showed defasciculation were categorized as strong. The size of the eye and the area covered by RGCs, as well as the areas of the optic tectum, forebrain and neuropil, were determined in *tg(Brn3c:mgfp)* control, *rtn4a-l* and *rtn4b* MO1-injected embryos, with 10 representative specimens at 3 and 5 dpf. Areas were measured in ImageJ software (National Institutes of Health, Bethesda, MD, USA) by using ventral and dorsal z-plane projections of the head. Data are represented as mean values, and error bars indicate the standard error of the mean. Data were analyzed using analysis of variance (ANOVA) and paired *t*-test were used after determining whether the sample datasets conform to a normal distribution. P-values are indicated as follows: *P ≤ 0.05. **P ≤ 0.01.

## Abbreviations

brn3c: Brain-specific homeobox/POU domain protein 3C (that is, POU domain, class 4, transcription factor 3 (pou4f3)); CNS: Central nervous system; DAPI: 4′,6-diamidino-2-phenylindole; dpf: Days postfertilization; GFP: Green fluorescent protein; hb9: Homeobox gene *hb9* (that is, motor neuron and pancreas homeobox 1 (*mnx1*)); hpf: Hours postfertilization; isl1: LIM homeobox gene *islet1*; LTP: Long-term potentiation; MO: Morpholino; NgR: Nogo receptor; PBS: Phosphate-buffered saline; PFA: Paraformaldehyde; PNS: Peripheral nervous system; RGC: Retinal ganglion cell; RHD: Reticulon homology domain; RTN: Reticulon; shh: *Sonic hedgehog*; tg: transgenic.

## Competing interests

The authors declare that they have no competing interests.

## Authors’ contributions

CAOS and EMT conceived of and designed the study. APO, CW, HA and EMT carried out the experiments. CAOS, EMT, APO and CW analyzed and interpreted the data. CAOS, EMT, APO, CW and HA contributed to the writing of the manuscript or to revising it critically for important intellectual content. All authors read and approved the final manuscript.

## Supplementary Material

Additional file 1 **Toxicity of *rtn4a *** **morpholino-mediated knockdown. (A)** Impaired development and viability after injection of *rtn4*-morpholino (MO). Embryos were microinjected at cell stages 1 to 4 and analyzed at 6 hpf and 15 hpf. Higher doses of *rtn4a*-MO resulted in delayed gastrulation at 6 hpf and reduced viability at 15 hpf compared to control embryos (arrowhead). **(B)***rtn4a* and *rtn4b* MOs were microinjected at 2.5 or 5.0 ng. Mortality, development and viability were evaluated for all experimental groups in at least three experiments. Embryos showing a curly tail phenotype upon *Rtn4a* knockdown were excluded from the experimental group. **(C)** Quantification of embryos with reduced anterior brain structures was carried out after knockdown of each Rtn4a isoform. Control (n = 95), *rtn4a*-l MO1 (*n* = 78), *rtn4a-m* MO1 (*n* = 23), *rtn4a-n* MO1 (*n* = 33) and *rtn4a* (*l, m* and *n*) MO1 (*n* = 78). **(D)** A second *rtn4b*-MO sequence (*rtn4b*-MO2) elicited the same phenotype as the *rtn4b*-MO1 sequence. At 15 hpf, the forebrain was flattened and the sizes of the head and eye anlage were reduced. At 1 dpf, *rtn4b*-MO2-injected embryos were smaller than control embryos, with reduced heads and eyes. Embryos also had an abnormally curved notochord. At 2 dpf, *rtn4b* morphants had shortened fore- and midbrain regions. At 3 dpf, *rtn4b* morphants developed a thinner tail, which curved ventrally, lacked lower jaws and had an inflated heart cavity. At 5 dpf, they did not recover any of the abnormalities seen at 3 dpf. **(E)** Acetylated tubulin staining of 2-dpf embryos injected with 5 ng of *rtn4*a MO revealed pathfinding mistakes of the lateral line. **(F)** Proportion of embryos with aberrant pathfinding of the lateral line at 2 dpf after injection of a mixture of 2 ng (*n* = 45) or 5 ng (*n* = 75) of each isoform of *rtn4a*-MO (arrow). Click here for file

Additional file 2: Table S1 Morpholino effect in retinal ganglion cells under different *rtn4* MO concentrations. Click here for file

Additional file 3 **Immunostaining of morphant embryos confirms the specificity of Rtn4a and Rtn4b antibodies. (A)** In 1 dpf control morpholino-injected embryos, the Rtn4a antibody labeled neural structures such as the neural tube. Upon morpholino knockdown of all *rtn4a* isoforms **(C)** or the *rtn4a-l* isoform only **(E)**, the signal appeared clearly reduced. Similarly, at 2 dpf, labeling of retinal ganglion cells (RGCs) and optic nerves (arrow) in control embryos **(B)** was reduced after knockdown of all or only the *rtn4a-l* isoforms **(D)** and **(F)**. **(G)** and **(H)** In control embryos, antibodies against Rtn4b labeled RGCs (arrow) **(G)**, spinal cord (arrow) and motor neurons (arrowheads) **(H)**. The signal in these structures was drastically reduced after Rtn4b downregulation **(I)** and **(J)**. (A), (C) and (F) show dorsal views (rostral to the left). (B), (D), (F), (G) and (I) show ventral views (rostral at the top). (H) and (J) show lateral views (rostral to the left). Click here for file

Additional file 4 **R** ***tn4b *** **morphants in *brn3c:mGFP *** **transgenic embryos. (A)** Branching of motor neurons in *rtn4b* morphants. Axonal projections (trunk segments 5 to 8 and 15 to 18) were analyzed. In mild phenotypes, motor axons showed misbranching and pathfinding mistakes, whereas in strong phenotypes defasciculation was also observed. **(B)** Proportion of abnormal motor axons in anterior and posterior segments in *rtn4b* morphants and in the rescue group at 2 dpf. *rtn4b* MO1 (*n* = 19), *rtn4b*-MO1 and *rtn4b*-mRNA (n = 25) and *rtn4b*-mRNA (*n* = 20). **(C)** Proportion of nonmotile embryos at 3 dpf in *rtn4b* morphants, rescued and *rtn4b-mRNA*-injected groups. Click here for file

Additional file 5 **Apoptosis in rtn4 morphant embryos.** Comparison of apoptosis in control **(A)**, *rtn4a* morphant **(B)** and *rtn4b* morphant **(C)**. Cell death was visualized at 1 dpf by acridine orange staining. **(D)** and **(E)** Quantification of acridine orange intensity in selected areas of the midbrain (square) and hindbrain (rectangle) showing increased staining (arrow) in both morphants. Control MO (5.0 ng) (*n* = 30), *rtn4a-l*-MO1 (5.0 ng) (*n* = 25) and *rtn4b*-MO1 (5.0 ng) (*n* = 24). Click here for file

Additional file 6 ***In vivo *** **bromodeoxyuridine labeling of 1- and 3-day postfertilization *rtn4 *** **morphant embryos. (A)** Overview of the bromodeoxyuridine (BrdU) pulse chase experiment. To maximize the labeling of cells entering the S-phase, a 1-hour BrdU pulse was applied at 1 dpf. Half of the embryos were fixed immediately after the pulse **(B)**, **(D)**, **(F)**, **(J)**, **(K)** and **(L)**, and the other half were fixed 2 days later **(C)**, **(E)**, **(G)**, **(M)**, **(N)** and **(O)**. Confocal maximum projections of midbrain and hindbrain sections showed a considerable amount of BrdU-labeled cells at 1 dpf (green) (B), (D), (F), (J), (K) and (L). Nuclei were counterstained with 4′,6-diamidino-2-phenylindole (DAPI) (red). At 1 dpf, the presumptive tectum of *rtn4a* morphants (D), especially *rtn4b* morphants (F), is reduced in size relative to control embryos (B). (C), (E), (G), (M), (N) and (O) BrdU retention in the tectum and hindbrain at 3 dpf. In addition to the reduced tectum in 1-dpf *rtn4a* and *rtn4b* morphants, cells in the 3-dpf *rtn4b* morphants show strong BrdU signaling 2 days after the BrdU chase (G) and (O). Only weak and diffused BrdU signaling was detected in *rtn4a* (E) and (N) and control (C) and (M) groups. (H) and (P) Quantification of total cells (red) and proliferating cells (green) in the tectum and hindbrain at 1 dpf in *rtn4a* and *rtn4b* morphants and the control group. (I) Quantification of total cells in the tectum at 3 dpf (red). (Q) Quantification of total (red) vs. BrdU-positive cells (yellow) in analyzed areas of the hindbrain at 3 dpf in *rtn4a* and *rtn4b* morphants and the control group.*Melanocytes. Control (1 dpf; *n* = 8), *rtn4a* (*n* = 11), *rtn4b* (*n* = 12), control 2 dpf (*n* = 6), *rtn4a* (n = 6) and *rtn4b* (*n* = 14). Scale bar = 50 μm. Click here for file
