# Supplementary figures and images for: Essential roles of zebrafish rtn4/Nogo paralogues in embryonic development
Source: Neural Dev. 2014 Apr 23;9:8. doi: 10.1186/1749-8104-9-8 (PMC4113184; doi:10.1186/1749-8104-9-8)

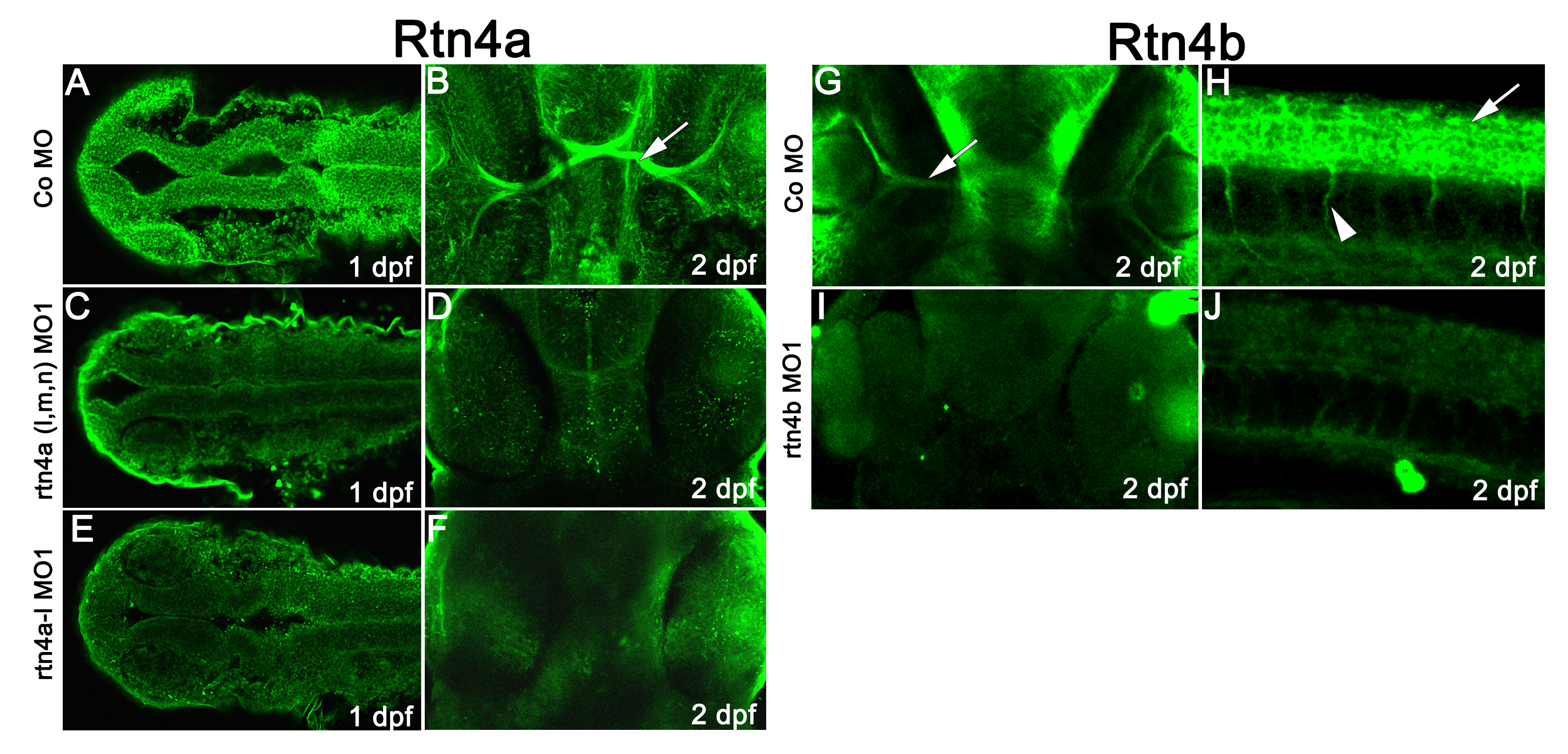

Supplement: Additional file 3 — Immunostaining of morphant embryos confirms the specificity of Rtn4a and Rtn4b antibodies. (A) In 1 dpf control morpholino-injected embryos, the Rtn4a antibody labeled neural structures such as the neural tube. Upon morpholino knockdown of all rtn4a isoforms (C) or the rtn4a-l isoform only (E), the signal appeared clearly reduced. Similarly, at 2 dpf, labeling of retinal ganglion cells (RGCs) and optic nerves (arrow) in control embryos (B) was reduced after knockdown of all or only the rtn4a-l isoforms (D) and (F). (G) and (H) In control embryos, antibodies against Rtn4b labeled RGCs (arrow) (G), spinal cord (arrow) and motor neurons (arrowheads) (H). The signal in these structures was drastically reduced after Rtn4b downregulation (I) and (J). (A), (C) and (F) show dorsal views (rostral to the left). (B), (D), (F), (G) and (I) show ventral views (rostral at the top). (H) and (J) show lateral views (rostral to the left). [file 1749-8104-9-8-S3.png]

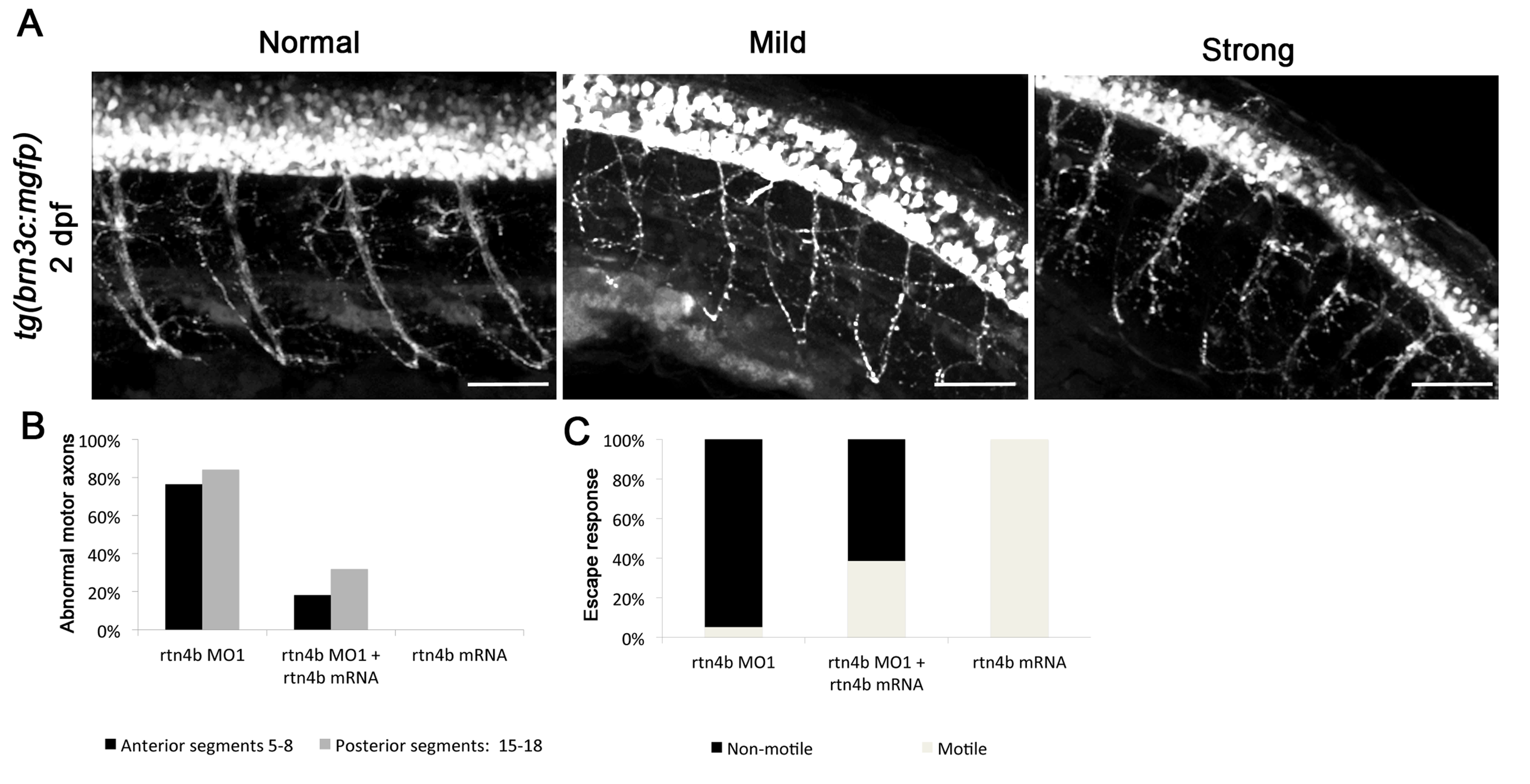

Supplement: Additional file 4 — R tn4b morphants in brn3c:mGFP transgenic embryos. (A) Branching of motor neurons in rtn4b morphants. Axonal projections (trunk segments 5 to 8 and 15 to 18) were analyzed. In mild phenotypes, motor axons showed misbranching and pathfinding mistakes, whereas in strong phenotypes defasciculation was also observed. (B) Proportion of abnormal motor axons in anterior and posterior segments in rtn4b morphants and in the rescue group at 2 dpf. rtn4b MO1 (n = 19), rtn4b-MO1 and rtn4b-mRNA (n = 25) and rtn4b-mRNA (n = 20). (C) Proportion of nonmotile embryos at 3 dpf in rtn4b morphants, rescued and rtn4b-mRNA-injected groups. [file 1749-8104-9-8-S4.png]

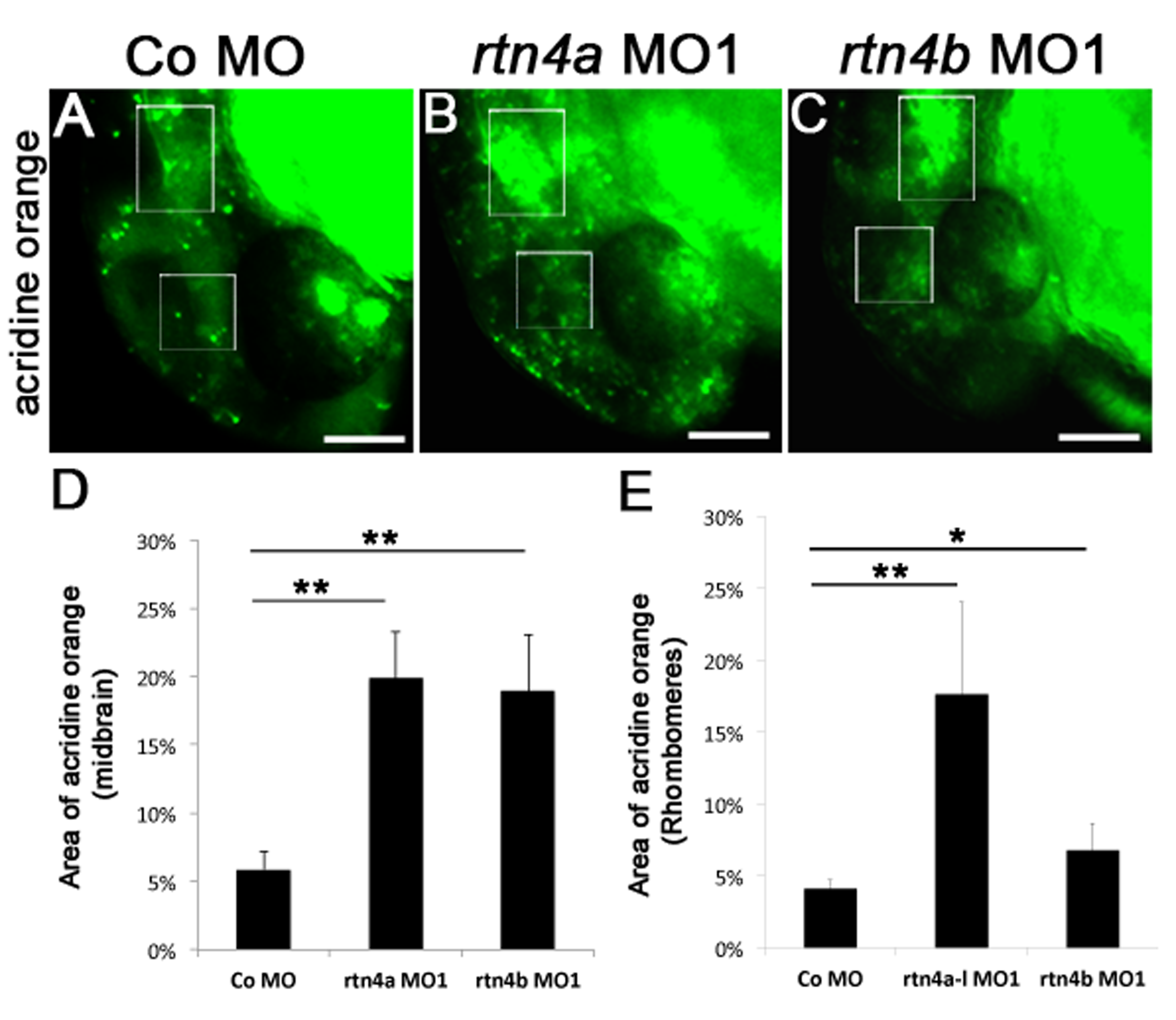

Supplement: Additional file 5 — Apoptosis in rtn4 morphant embryos. Comparison of apoptosis in control (A), rtn4a morphant (B) and rtn4b morphant (C). Cell death was visualized at 1 dpf by acridine orange staining. (D) and (E) Quantification of acridine orange intensity in selected areas of the midbrain (square) and hindbrain (rectangle) showing increased staining (arrow) in both morphants. Control MO (5.0 ng) (n = 30), rtn4a-l-MO1 (5.0 ng) (n = 25) and rtn4b-MO1 (5.0 ng) (n = 24). [file 1749-8104-9-8-S5.png]

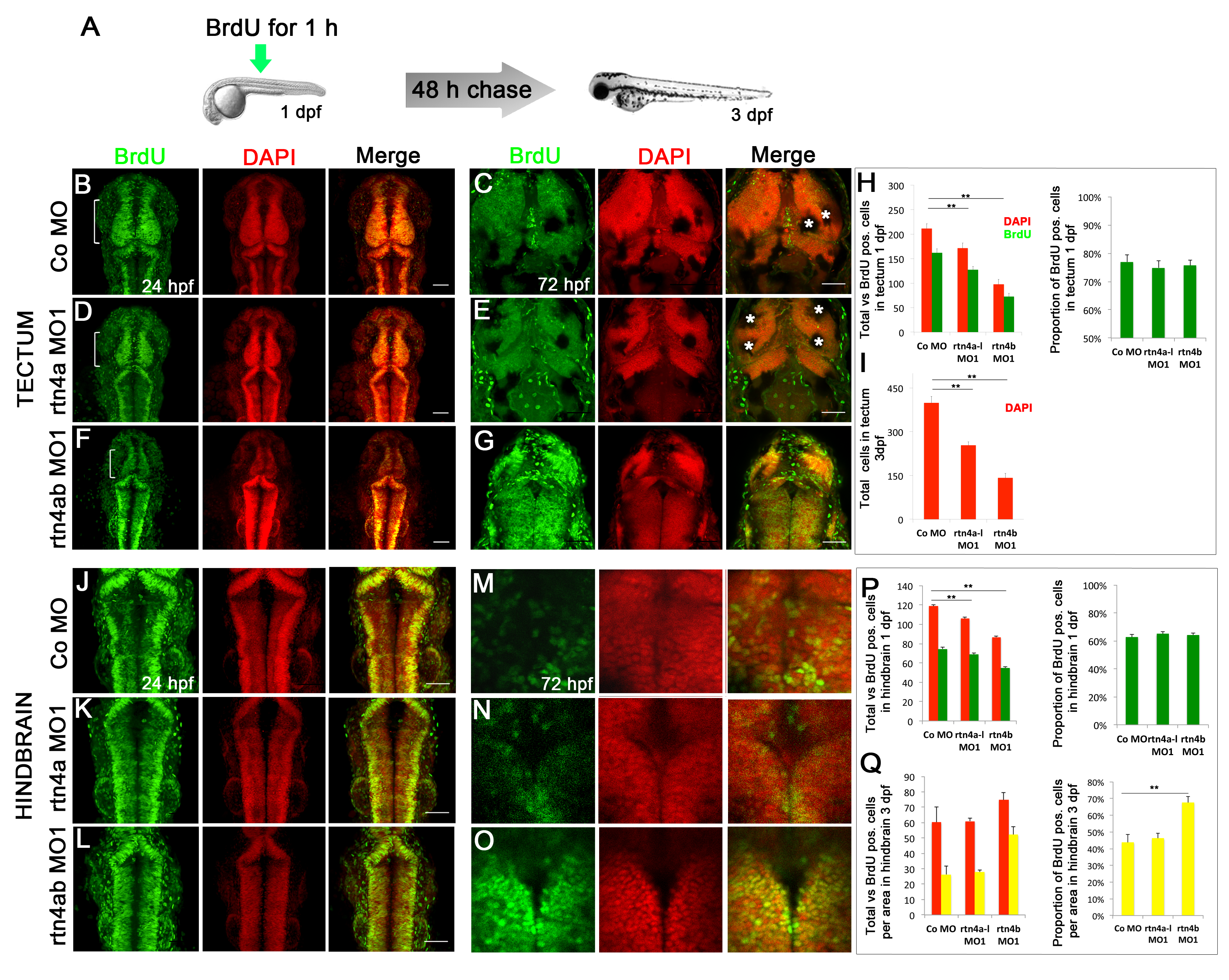

Supplement: Additional file 6 — In vivo bromodeoxyuridine labeling of 1- and 3-day postfertilization rtn4 morphant embryos. (A) Overview of the bromodeoxyuridine (BrdU) pulse chase experiment. To maximize the labeling of cells entering the S-phase, a 1-hour BrdU pulse was applied at 1 dpf. Half of the embryos were fixed immediately after the pulse (B), (D), (F), (J), (K) and (L), and the other half were fixed 2 days later (C), (E), (G), (M), (N) and (O). Confocal maximum projections of midbrain and hindbrain sections showed a considerable amount of BrdU-labeled cells at 1 dpf (green) (B), (D), (F), (J), (K) and (L). Nuclei were counterstained with 4′,6-diamidino-2-phenylindole (DAPI) (red). At 1 dpf, the presumptive tectum of rtn4a morphants (D), especially rtn4b morphants (F), is reduced in size relative to control embryos (B). (C), (E), (G), (M), (N) and (O) BrdU retention in the tectum and hindbrain at 3 dpf. In addition to the reduced tectum in 1-dpf rtn4a and rtn4b morphants, cells in the 3-dpf rtn4b morphants show strong BrdU signaling 2 days after the BrdU chase (G) and (O). Only weak and diffused BrdU signaling was detected in rtn4a (E) and (N) and control (C) and (M) groups. (H) and (P) Quantification of total cells (red) and proliferating cells (green) in the tectum and hindbrain at 1 dpf in rtn4a and rtn4b morphants and the control group. (I) Quantification of total cells in the tectum at 3 dpf (red). (Q) Quantification of total (red) vs. BrdU-positive cells (yellow) in analyzed areas of the hindbrain at 3 dpf in rtn4a and rtn4b morphants and the control group.*Melanocytes. Control (1 dpf; n = 8), rtn4a (n = 11), rtn4b (n = 12), control 2 dpf (n = 6), rtn4a (n = 6) and rtn4b (n = 14). Scale bar = 50 μm. [file 1749-8104-9-8-S6.png]
